# The role of carbonic anhydrase VI in bitter taste perception: evidence from the *Car6*^−/−^ mouse model

**DOI:** 10.1186/s12929-014-0082-2

**Published:** 2014-08-19

**Authors:** Maarit Patrikainen, Peiwen Pan, Natalia Kulesskaya, Vootele Voikar, Seppo Parkkila

**Affiliations:** 1 School of Medicine and BioMediTech, University of Tampere, Tampere FI-33014, Finland; 2 Neuroscience Center, University of Helsinki, Helsinki FI-00014, Finland; 3 Fimlab Ltd and Tampere University Hospital, Tampere FI-33520, Finland

**Keywords:** Bitter, Carbonic anhydrase, Gustin, Mouse, Saliva, Taste

## Abstract

**Background:**

Carbonic anhydrase VI (CA VI) is a secretory isozyme of the α-CA gene family. It is highly expressed in the salivary and mammary glands and secreted into saliva and milk. Although CA VI was first described as a gustatory protein, its exact functional roles have remained enigmatic. Interestingly, polymorphism of the *CA6* gene was recently linked to bitter taste perception in humans. In this study, we compared the preference of *Car6*^−/−^ and wild-type mice for different taste modalities in an IntelliCage monitoring environment. Morphologies of taste buds, tongue papillae, and von Ebner’s glands were evaluated by light microscopy. Cell proliferation and rate of apoptosis in tongue specimens were examined by Ki67 immunostaining and fluorescent DNA fragmentation staining, respectively.

**Results:**

The behavioral follow up of the mice in an IntelliCage system revealed that *Car6*^−/−^ mice preferred 3 μM quinine (bitter) solution, whereas wild type mice preferred water. When the quinine concentration increased, both groups preferentially selected water. Histological analysis, Ki67 immunostaining and detection of apoptosis did not reveal any significant changes between tongue specimens of the knockout and wild type mice.

**Conclusions:**

Our knockout mouse model confirms that CA VI is involved in bitter taste perception. CA VI may be one of the factors which contribute to avoidance of bitter, potentially harmful, substances.

## Background

Carbonic anhydrase VI (CA VI) is the only secretory isozyme in the α-CA enzyme family. In the first report on CA VI Henkin’s group identified a novel protein, gustin, from human saliva [1], which was later shown to be identical to CA VI [2]. Independently, this same protein was described as a distinct CA enzyme by Fernley’s team, who identified a novel high molecular weight form of CA in the sheep parotid gland and saliva [3]. Later, CA VI was also isolated from rat [4] and human saliva [5]. Immunohistochemical studies have indicated that CA VI is highly expressed in the serous acinar cells of the parotid and submandibular glands [6],[7]. In fact, it is one of the major protein constituents of human saliva. The mean concentration of CA VI in paraffin-stimulated saliva is 6.8 +/− 4.3 mg/L and the secretion rate is 10.2 +/− 7.9 μg/min [8]. Secretion of CA VI is tightly regulated by circadian periodicity; the levels are low during the night and increase rapidly after awakening [9]. The expression of CA VI is not restricted only to the salivary glands. Ogawa’s group demonstrated CA VI expression in the lacrimal gland [10], and Karhumaa and coworkers found that milk contains high levels of secretory CA VI [11]. The 42-kDa polypeptide purified from human milk by CA inhibitor affinity chromatography shared 100% homology with salivary CA VI according to a protein sequence analysis. A time-resolved immunofluorometric assay showed that colostrum contained an eight times higher concentration of CA VI than mature milk, the latter containing concentrations comparable to the mean levels in saliva.

Even though CA VI was originally discovered more than 30 years ago, its physiological role has remained unclear. As an enzymatically active CA it could maintain optimal pH homeostasis within the oral cavity and upper alimentary tract [12],[13]. The high concentrations in milk and colostrum suggested that it could participate in the developmental processes of the gastrointestinal canal during the postnatal period [11]. Earlier studies also suggested that CA VI present in saliva could play a protective role against cariogenesis [14]. These and other possible physiological roles can now be studied using the recently described knockout mouse model for CA VI deficiency [15]. The knockout mice are viable, fertile, and have shown a normal life span. Surprisingly, an *in vivo* cariogenesis model revealed that the CA VI knockout mice had a lower rate of cariogenesis and oral colonization of *Streptococcus mutans* than the wild type (WT) controls [16]. Histological analyses have indicated a greater number of lymphoid follicles in the small intestinal Peyer’s patches of the knockout mice, suggesting an immunological phenotype for CA VI deficiency [15]. This was further supported by functional clustering of differentially expressed genes, which revealed a number of altered biological processes in the knockout mice. Importantly, these included a Gene Ontology (GO) term for a biological process called “immune system process” in the duodenum.

Henkin’s group linked gustin (CA VI) to the regulation of taste function in 1981 [17]. They found that the biochemical characteristics of CA VI were similar in protein isolated from both subjects with normal taste acuity and from patients with hypogeusia. Interestingly, they reported that hypogeusic subjects had salivary CA VI concentration as low as 20% that of normal subjects, but they did not link CA VI to any specific taste modality. Recently, Barbarossa’s group has shown a link between bitter taste modality and CA VI by finding polymorphism in the *CA6* gene (rs2274333 (A/G)) which contributes to 6-*n*-propylthiouracil taster status [18]. Later, they elegantly showed that alterations of bitter taste function are due to polymorphic changes in both bitter receptor gene (*TAS2R38*) and *CA6* gene, while also requiring contributions from other still unknown factors [19]. Most recently, Barbarossa’s group demonstrated that rs2274333 polymorphic change in the *CA6* gene affects 6-*n*-propylthiouracil sensitivity by acting on fungiform papilla development and maintenance [20]. There is still another important piece of evidence that CAs indeed contribute to taste perception. Chandrashekar and coworkers demonstrated by targeted genetic ablation and neurophysiological measurements, that sour-sensing cells act as taste sensors for carbonation, and also showed that membrane-associated CA IV functions as the principal CO_2_ taste sensor [21].

In this study, we investigated the role of CA VI in taste function by utilizing the CA VI-deficient mouse model. These mice were placed in an IntelliCage system [22] for automated behavioral screening and their preferences for various taste modalities were analyzed. The results provide a mouse model confirmation that CA VI deficiency leads to abnormal bitter taste perception.

## Methods

### Ethical approval

The production of the knockout mouse line was approved by the Animal Experimentation Committee of the University of Oulu, Finland. Behavioural experiments were carried out in accordance with the Guidelines laid down with the European Communities Council Directive of 24 November 1986 (86/609/EEC) and were approved by the County Administrative Board of Southern Finland (license number ESAVI-2010-09011/Ym-23).

### *Car6*^−/−^ mice

Generation and phenotypic characterization of *Car6*^−/−^ mice have been described previously by Pan et al. [15]. The mice with the targeted allele were backcrossed more than 10 generations to obtain mice with a pure C57BL/6 strain background. *Car6*^*−/−*^ mice proved to be fertile and showed normal life-span. The absence of CA VI protein in saliva and salivary glands was confirmed by immunohistochemistry and western blotting.

### Behavioral monitoring

#### *Animals*

Nine WT female and nine knock-out female mice at the age of 12 weeks, at the beginning of experiment, were used for behavioral analysis. One week before onset of testing in an IntelliCage, RFID transponders (Planet ID GmbH, Essen, Germany) were injected subcutaneously in the dorso-cervical region under isoflurane inhalation anesthesia. Throughout the experiment the mice were maintained under 12/12 h light cycle (lights on at 06:00) at controlled temperature (21 ± 1°C) and humidity (50–60%).

#### Apparatus and procedure

The IntelliCage apparatus (NewBehavior AG, Zurich, Switzerland, www.newbehavior.com) is placed in a polycarbonate cage (20.5 cm high, 58 × 40 cm top, 55 × 37.5 cm bottom, Tecniplast, 2000P, Buguggiate, Italy) and accommodates up to 16 mice. Its aluminum top contains a freely accessible food rack filled with standard mouse chow (Teklad 2016, Harlan). The floor is covered with bedding (aspen chips 5 × 5 × 1 mm, Tapvei Oy, Finland) and provides four central red shelters (Tecniplast, Buguggiate, Italy). Four triangular conditioning chambers (15 × 15 × 21 cm) are fitted in the cage corners and each provide room for one mouse at a time. Each chamber contains two drinking bottles, accessible via two round openings (13 mm diameter) with motorized doors. Three multicolor LEDs are mounted above each door and the chamber ceiling contains a motorized valve for delivery of air puffs. Mice that access a chamber are identified by a circular RIFD antenna at its entrance (30 mm inner diameter) and the duration of their visit is determined by both the antenna reading and a temperature sensor that detects the presence of the animal inside the corner. During a visit, number and duration of individual nosepokes at each door are recorded using IR-beam sensors. Licking episodes at each bottle are monitored using lickometers (duration of the episode, number of licks, total contact time). IntelliCages have individual controllers and are connected to a central PC running the IntelliCage Plus software that permits designing and running experiments, as well as analysis of the recorded data (IntelliCage Plus, NewBehavior AG).

The animals were habituated to the new environment for 8 days. During this period, every visit to the chamber opened both doors for 7 sec allowing access to drinking bottles filled with tap water. For testing spontaneous taste preference, one corner contained two bottles with tap water. The other three corners contained bottles with different concentrations of taste solution (one concentration per corner). Each taste modality was tested over 4 days, and the position of bottles was changed every day in order to avoid development of place preference or avoidance. Four taste modalities were tested in the following order and concentrations: 1) Sweet – saccharin 0.1, 5, 50 g/l; 2) Salty – NaCl 50, 150, 450 mM; 3) Sour – citric acid 0.1, 10, 100 mM; 4) Bitter – quinine 0.003, 0.03, 0.3 mM. Two-day wash-out periods with tap water in all corners and bottles were applied before subsequent tastants. The number of licks in each corner was recorded and used for calculating the preference expressed as a percentage of licks at different concentrations. The data were compared between genotypes by repeated measures ANOVA with preference to tastant as a within-subject factor, followed by Newman-Keuls post hoc test.

### Morphological analyses and immunohistochemistry

Whole tongue samples were taken from the wild-type (n = 9) and *Car6*^*−/−*^ (n = 9) adult mice. The samples were dissected, fixed in 4% formaldehyde overnight, embedded in paraffin, and sectioned to a thickness of 5 μm. For morphological analysis paraffin was removed with xylene, rehydrated by a descending series of ethanol, and the sections were stained with hematoxylin and eosin. Morphologies of different papillae (circumvallate, fungiform, and filiform) and von Ebner’s glands were evaluated by light microscopy. The numbers of fungiform papillae were evaluated in 27 sections/group (three sections/mouse). The anterior half of the tongue was examined for the presence of fungiform papillae. The number of fungiform papillae from each section were counted. Because of the small sample size, and potential non-normal distribution of section cell counts, the Mann–Whitney test was used for comparing the two groups. P value < 0.05 was considered statistically significant.

Immunohistochemistry for the proliferation of marker Ki67 was performed using a Vectastain Elite ABC kit (Vector Laboratories, Burlingame, CA, USA) following the manufacturer’s instructions. Prior to staining, the sections were boiled in 0.01 M sodium citrate buffer, pH 6.0, for 20 min. Endogenous peroxidase activity was blocked by treatment with 3% H_2_O_2_ solution. Nonspecific binding of the primary antibody was prevented using 10% normal rabbit serum (NRS) as a blocking agent. The sections were incubated overnight at + 4°C in a primary antibody solution containing monoclonal rat anti-mouse Ki67 (Dako Denmark A/S, Glostrup, Denmark) (diluted 1:100), 1% NRS, and 0.1% Tween-20 (Sigma-Aldrich, St. Louis, MO, USA) in phosphate buffered saline (PBS). Biotinylated rabbit anti-rat serum (Vector Laboratories) (diluted 1:1000) was used as a secondary antibody, and the sections were incubated 30 min at room temperature. DAB (3, 3’-diaminobenzidine, Invitrogen, Camarillo, CA, USA) was used for precipitating the substrate, and the sections were finally counterstained with Mayer’s hematoxylin. Positively stained cells were counted from one field/tongue photographed with 100× magnification (Nikon Microphot-FXA, Nikon Instruments Europe B.V., Amsterdam, Netherlands). To determine the difference between the two groups, 21 wild type and 16 knockout mice fields were analyzed. Statistical analysis was performed using Mann–Whitney test.

For studying the apoptotic level of tongue epithelial cells we used FragEL DNA Fragmentation Detection Kit, Fluorescent-TdT Enzyme (Calbiochem, EMD Chemicals, Inc., San Diego, CA, USA) according to manufacturer’s instructions. After deparaffinisation and rehydration the sections were incubated with proteinase K (20 μg/ml) at room temperature for 20 min. Prior to the equilibration step positive control sections were treated with 1 μg/μl DNase I in 1 mM MgSO_4_/1 × tris-buffered saline (TBS) and incubated for 20 min at room temperature. After 15 min incubation in 1 × TdT Equilibration buffer, the sections were covered with TdT Labeling Reaction Mixture. Negative control was prepared at labeling step using dH_2_O instead of enzyme in TdT Labeling Reaction mixture. Samples were incubated at + 37°C for 1.5 h. Sections were mounted with Fluorescein-FragEL mounting medium and photographed using Nikon Mikrophot-FXA microscope with 450–490 nm filter using 100 × magnification (Nikon Instruments Europe BV). Labeled nuclei were analyzed from 7 *Car6*^−/−^ and 7 WT mice tongues (two fields/tongue). Statistical comparison between the two groups was performed using Mann–Whitney test.

## Results

### Behavioral study

**Figure 1:**
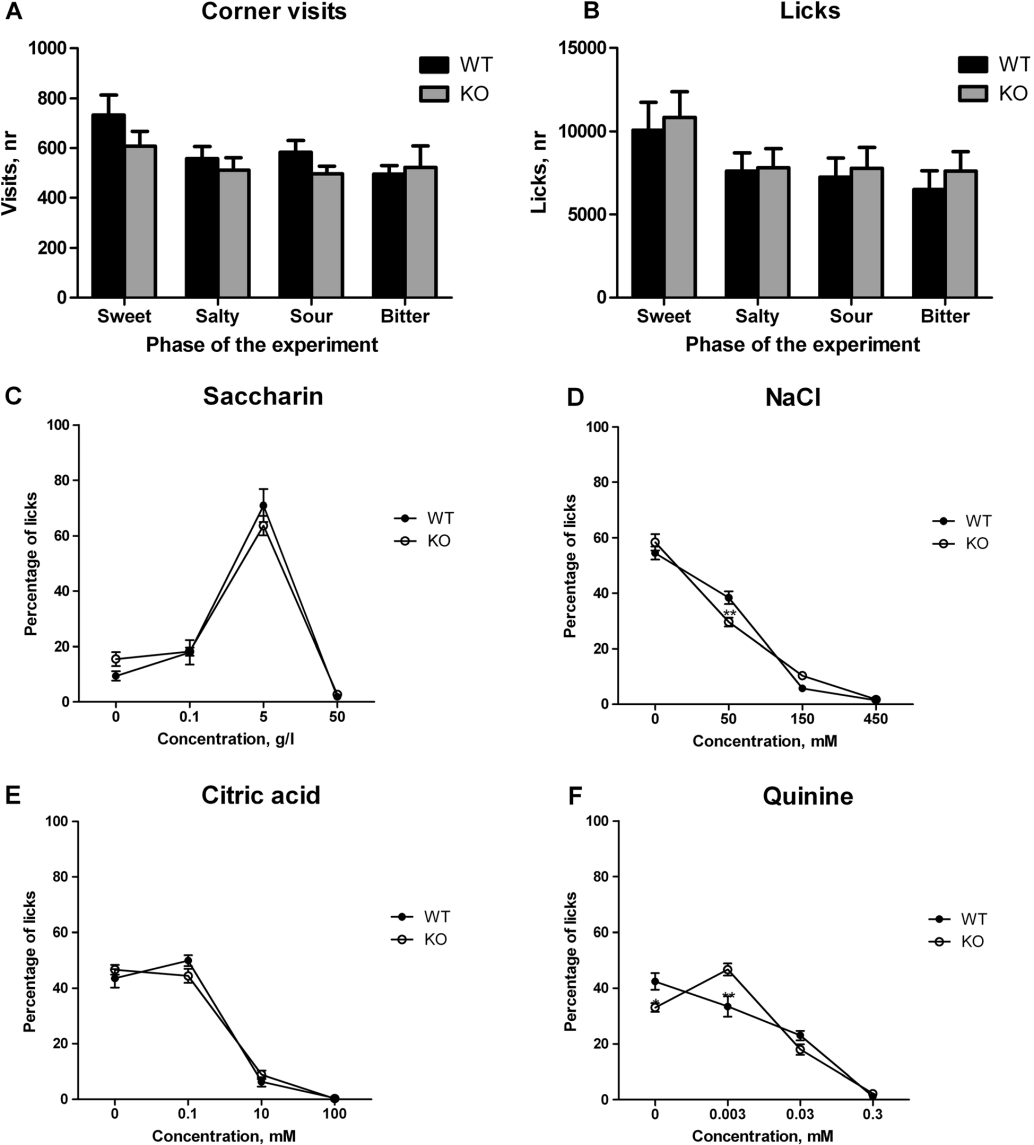
**Assessment of taste preference in the IntelliCage. A**. Total number of corner visits during 4 days of each phase of the experiment. **B**. Total number of licks during 4 days of each phase of the experiment. **C**. Percentage of licks of sweet (saccharin) solution at different concentrations. **D**. Percentage of licks of salty (NaCl) solution at different concentrations. **E**. Percentage of licks of sour (citric acid) solution at different concentrations. **F**. Percentage of licks of bitter (quinine) solution at different concentrations. * - p < 0.05, ** - p < 0.01 between the genotypes; # - p < 0.01 compared to water.

There was no difference in body weight between the WT and *Car6*^*−/−*^ mice. Moreover, the activity (number of corner visits, Figure 1A) and total liquid consumption (number of licks, Figure 1B) were similar between the groups in all phases of the experiment.

The mice showed similar preference for saccharin 5 g/l, whereas 50 g/l was avoided by both groups (Effect of concentration F(3,48) = 126.9, p < 0.0001; no significant interaction between genotype and concentration F(3,48) = 1.1, p = 0.34) (Figure 1C). Both groups displayed a similar preference for water and avoidance of 150 and 450 mM of NaCl. However, Car6^−/−^ mice showed slightly lower percentage of licks at 50 mM NaCl as compared with WT mice (effect of concentration F(3,48) = 300.2, p < 0.0001; significant interaction between genotype and concentration F(3,48) = 4.5, p = 0.0075) (Figure 1D). Both groups showed equal preference for water and 0.1 mM of citric acid whereas 10 mM and 100 mM concentrations of citric acid were avoided (effect of concentration F(3,48) = 246.6, p < 0.0001; no significant interaction between genotype and concentration F(3,48) = 1.6, p = 0.21) (Figure 1E). When testing bitter taste, *Car6*^−/−^ mice showed preference for 0.003 mM quinine solution, whereas WT mice preferred water (Figure 1F). Higher concentrations of quinine were clearly avoided by both WT and knockout mice (effect of concentration F(3,48) = 103.4, p < 0.0001; significant interaction between genotype and concentration F(3,48) = 7.8, p = 0.0002).

### Tongue morphology, cell proliferation and apoptosis

**Figure 2:**
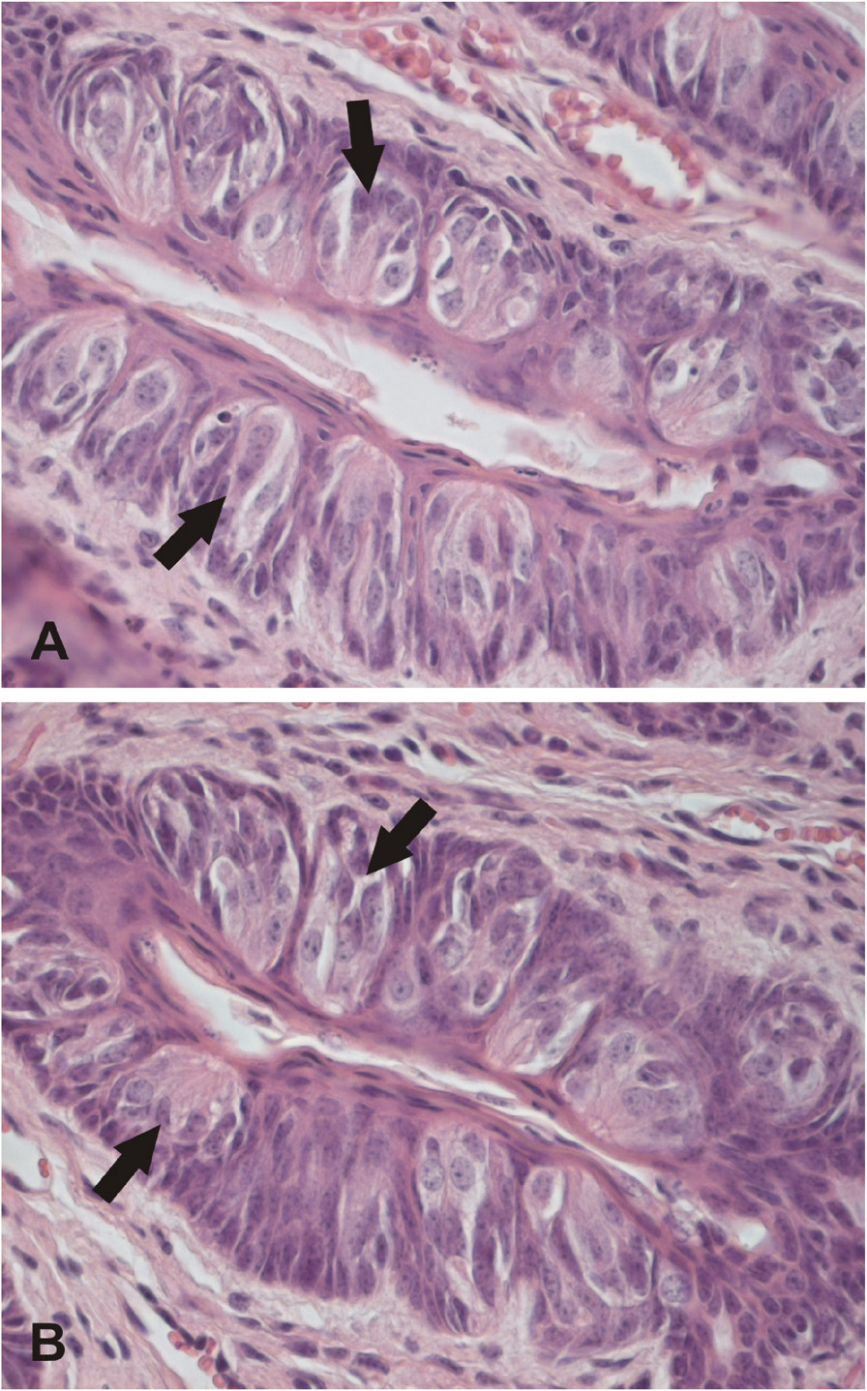
**Histological images of tongue morphology in*Car6***^**−/−**^**(A) and WT (B) mice.** Arrows point to some examples of taste buds. No significant differences are visible in morphology. Original magnifications x 400.

Tongue histologies were analyzed from *Car6*^−/−^ and WT mice, including the morphology and number of taste buds and different types of papillae. Figure 2 shows representative images of morphology. The morphologies of three different papillae (circumvallate, fungiform and filiform) were not significantly different between the two groups of mice, nor did we find any changes in the von Ebner’s glands (data not shown). The number of fungiform papillae showed no change in *Car6*^−/−^ mice as compared to the control mice (p = 0.58, Mann–Whitney test).

**Figure 3:**
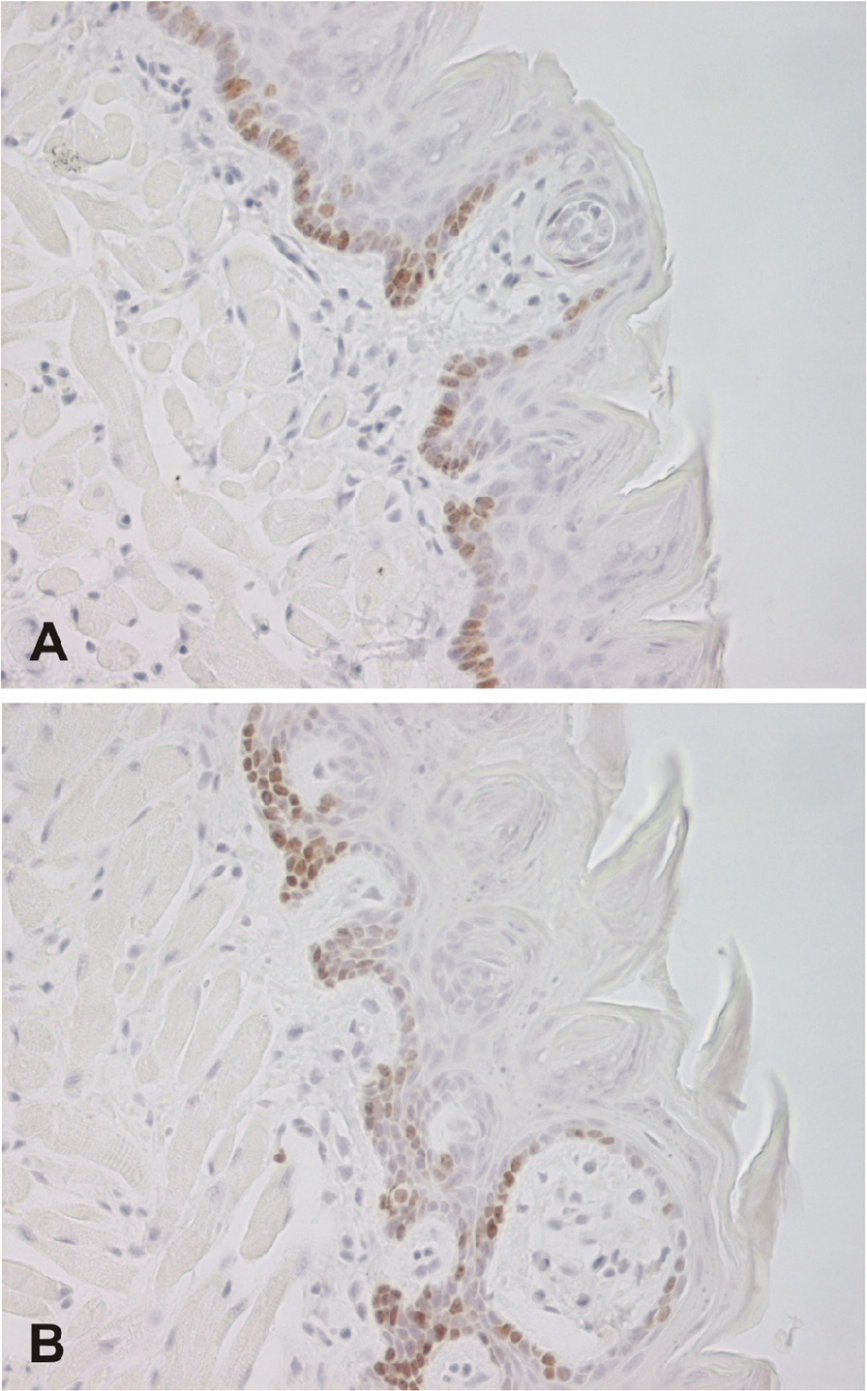
**Immunohistochemical staining of Ki67 proliferation marker in tongue specimens from*Car6***^**−/−**^**(A) and WT (B) mice.** Both samples demonstrate a number of positive nuclei mainly located in the basal layer of the stratified epithelium. Original magnifications × 400.

Immunohistochemistry for the proliferation marker Ki67 showed positive for nuclei in the tongue epithelium of all specimens, as expected. Mean counts for cell proliferation were 105.3/section (range 35–159) and 100.2/section (range 8–168) in the *Car6*^−/−^ and WT mice, respectively. No statistically significant difference was observed between the *Car6*^−/−^ and WT mice (p = 0.63, Mann–Whitney test) (Figure 3). Rate of apoptosis in the tongue specimens was analyzed using a DNA fragmentation detection kit. Mean values for the apoptosis count were 2.9/section (range 0–9) and 2.0/section (range 0–5) in the *Car6*^−/−^ and WT mice, respectively. No statistically significant difference was found in the rate of apoptosis between the *Car6*^−/−^ and WT mice (p = 0.69, Mann–Whitney test).

## Discussion

The present data shows that CA VI deficiency results in an altered behavior in mice for preferred taste. The most significant change was observed in bitter taste, which is well in line with the recent observations from Barbarossa’s group, showing an altered bitter taste perception in subjects with *CA6* gene polymorphism [18]-[20]. The previous study indicated that both *TAS2R38* and *CA6* loci are important for discriminating low concentrations of the bitter tasting chemical, 6-n-propylthiouracil [19]. In our study, the CA VI deficient mice showed a lower percentage of licks at 50 mM NaCl than the WT mice, suggesting that CA VI is somehow involved in perception of salty taste. This finding has not been reported earlier and warrants further investigations. There were no other significant behavioral changes in terms of the taste preference.

It is commonly known that oral and perioral infections can cause taste dysfunction [23]. Henkin’s group demonstrated that in human patients suffering from taste dysfunction and influenza-like symptoms, the taste buds in circumvallate papillae exhibited apoptotic features, such as severe vacuolization and cellular degeneration [24]. These patients also had lowered concentrations of CA VI in their saliva. Treatment with zinc normalized the CA VI concentrations and the senses of taste and smell in some cases. Interestingly, the taste bud morphology was normalized in these patients, suggesting that CA VI may function as a trophic factor for the taste bud stem cells. Our analysis by light microscopy revealed no apparent changes in taste bud morphology in CA VI deficient mice nor did we observe any difference in the rate of apoptosis. This might suggest that the apoptotic changes observed earlier [24] are primarily due to the viral infection itself [25] rather than a result of CA VI deficiency. Similarly, the normalized taste bud morphology after the zinc treatment can be attributed to the effects of zinc on multiple cellular processes, other than those regulated by CA VI.

The present morphological analyses showed no significant change in the number of fungiform papillae in *Car6*^−/−^ mice compared to controls. This finding potentially contradicts the previous observations which showed an abnormal development of fungiform papillae in humans with *CA6* gene polymorphism [20]. The previous study also showed that CA VI protein induces proliferation of tongue epithelial cells. Even though our results were not able to confirm the role of CA VI in morphogenesis of fungiform papillae, nor did they show any effect in proliferation, the recent findings from Barbarossa’s group are attractive and warrant confirmation in different cell lines. Importantly, our *in vivo* studies were performed using a mouse model in which only a single salivary factor, CA VI, had been knocked out. There are a number of other growth factors present in saliva [26], and deficiency of CA VI alone may not be sufficient to affect significantly the cell proliferation rate. In spite of these negative results regarding the role of CA VI in cell proliferation, it is still attractive to speculate whether addition of exogenous CA VI into various products, such as mouth rinse, tooth paste, or chewing gum, could have some beneficial effects on oral health.

## Conclusions

Our results on *Car6*^−/−^ mice confirm that CA VI deficiency leads to abnormal bitter taste perception. This finding agrees well with the recent observations on the role of CA VI in human taste function. CA VI may be one of the factors, which can contribute to avoidance of bitter substances in mammals.

## Competing interests

The authors declare that they have no competing interests.

## Authors’ contributions

All authors were involved in design of the study. PP generated and characterized the *Car6*^−/−^ mouse colony. MP carried out the histochemical and immunohistochemical analyses. NK and VV performed the taste function tests by IntelliCage apparatus and analyzed the data. SP and VV drafted the manuscript. All authors read and approved the final manuscript.
